# ORM Promotes Skeletal Muscle Glycogen Accumulation via CCR5-Activated AMPK Pathway in Mice

**DOI:** 10.3389/fphar.2016.00302

**Published:** 2016-09-13

**Authors:** Zhen Qin, Jing-Jing Wan, Yang Sun, Peng-Yuan Wang, Ding-Feng Su, Hong Lei, Xia Liu

**Affiliations:** Department of Pharmacology, School of Pharmacy, Second Military Medical UniversityShanghai, China

**Keywords:** orosomucoid, CCR5, AMPK, glycogen, skeletal muscle

## Abstract

We found previously that acute phase protein orosomucoid reacts to fatigue and activates C-C chemokine receptor type 5 to increase muscle glycogen storage and enhance muscle endurance (Lei et al., 2016). To explore the underlying molecular mechanisms, we investigated the role of AMP-activated protein kinase, a critical fuel sensor in skeletal muscle, in C-C chemokine receptor type 5-mediated orosomucoid action. It was found orosomucoid increased skeletal muscle AMP-activated protein kinase activation in a time- and dose- dependent manner, which was largely prevented by pharmacological blocking or knockout of C-C chemokine receptor type 5. Administration of orosomucoid also significantly increased the de-phosphorylation and activity of muscle glycogen synthase, the rate-limiting enzyme for glycogen synthesis. The effect was largely absent in mice deficient in C-C chemokine receptor type 5^−/−^ or AMP-activated protein kinase α2^−/−^, the predominant isoform in skeletal muscle. Moreover, deletion of AMP-activated protein kinase α2 abolished the effect of orosomucoid on fatigue and muscle glycogen. These findings indicate that orosomucoid may promote glycogen storage and enhance muscle function through C-C chemokine receptor type 5-mdiated activation of AMP-activated protein kinase, which in turn activates glycogen synthase and increases muscle glycogen.

## Introduction

Orosomucoid (ORM) is an acute phase protein, with very low pI of 2.8–3.8 and a very high carbohydrate content of 45%. It is predominantly synthesized in the liver and many extra-hepatic tissues have also been reported to produce ORM under physiological and pathological conditions. Many biological activities of ORM have been identified including acting as a disease marker, modulating immunity, and regulating energy homeostasis. Its related receptor has been preliminarily explored in macrophages, neutrophils, and hypothalamus neurons, involving the membrane receptor CCR5, Siglect-5, and leptin receptor (LepR), respectively (Luo et al., 2015; Sun et al., 2016). It was found that ORM induces rises in cytosolic Ca^2+^ in neutrophil granulocytes via Siglec-5 to modulate leukocyte functions during an inflammatory process (Gunnarsson et al., 2007). Besides, the ORM is able to bind directly to LepR and activate the receptor-mediated JAK2–STAT3 signaling in hypothalamus tissue and GT1-7 cells to regulate food intake and energy homeostasis in response to nutrition status (Sun et al., 2016).

In our previous study, we found that the expression of ORM is markedly increased in response to various forms of fatigue, such as sleep deprivation, forced swimming, and treadmill running. Interestingly, ORM is able to act on cell membrane receptor CCR5 to increase muscle glycogen and enhance muscle endurance, representing a positive feedback mechanism to resist fatigue and maintain homeostasis (Yi et al., 2014; Lei et al., 2016). Noteworthily, the anti-fatigue action of ORM is completely abolished by CCR5 antagonist or knockout, indicating that CCR5 is the major mediator of the effect. However, the underlying signal pathway through which ORM-activated CCR5 promotes the glycogen storage remained unknown.

AMP-activated protein kinase (AMPK), a heterotrimeric complex composed of one catalytic (α) subunit and two regulatory subunits (β and γ) (Kahn et al., 2005; Dolinsky et al., 2015), is reported to be a key sensor of fuel and energy status in skeletal muscle, and is important for glucose uptake and glycogen storage in the myocytes (Hardie and Sakamoto, 2006; Hunter et al., 2011). Recent evidence showed that muscle AMPK played an important role in exercise. AMPKα2 is the predominant isoform contributing to AMPK activity in skeletal muscle. Mice specifically expressing an inactive form of AMPKα2 in skeletal muscle displayed exercise intolerance (Fujii et al., 2007), and mice with defective muscle AMPK, named lazy mice, showed a decrease in voluntary activity (Mu et al., 2001, 2003). Moreover, whole-body depletion of AMPKα2 was associated with a defect in glycogen synthesis (Andreelli et al., 2003), and acute or chronic activation of AMPK could increase the glycogen storage in skeletal muscle (Ojuka et al., 2000; Vitzel et al., 2001), suggesting a possible causal relationship between AMPK, glycogen synthesis, and exercise tolerance.

C-C chemokine receptor type 5 (CCR5) belongs to the superfamily of G-protein-coupled receptors (Lee et al., 2014), which contain seven transmembrane helices and transmit signals from extracellular signals to intracellular pathways through heterotrimeric G-proteins (de Munnik et al., 2015; Lohse, 2015). CCR5 is involved in the regulation of inflammation and immune response and also functions as a coreceptor for HIV to enter into cells. It is worth noting that CCR5 engagement increases glucose uptake and activates AMPK to accumulate ATP and meet the energy demands of chemotaxis in activated T cells (Chan et al, 2012). It was also found that AMPK mediates CCR5-activating ckemokine-induced cell migration in human chondrosarcoma (Hsu et al., 2013). Therefore, we ask whether AMPK is critical in the signal pathway from the ORM/CCR5 activation to glycogen synthesis in the skeletal muscles and mediates the anti-fatigue action of ORM.

## Materials and methods

### Reagents

ORM (Cat. No. G9885) was purchased from Sigma (St. Louis, MO). CCR5 antagonist Maraviroc was generously provided by Professor Xin Xie (Shanghai Institute of Materia Medica, CAS). Antibodies specific to AMPK (Cat. No. 2603), p-AMPK (at T172, Cat. No. 4188), glycogen synthase (GS, Cat. No. 3893), p-GS (Cat. No. 3891), and GAPDH (Cat. No. 2118) were obtained from Cell Signaling (Danvers, MA).

### Animals

C57BL/6 mice (18–22 g) were purchased from Sino-British SIPPR/BK Laboratory Animals (Shanghai, China). The CCR5-deficient mice (B6.129P2-Ccr5tm1Kuz/J, Stock Number: 005427) were obtained from Jackson Laboratory (Bar Harbor, MA). AMPKα2 knockout mice were generated by S. Vaulont as described previously (Viollet et al., 2003). Mice were 6–8 weeks of age at the start of the experiments. All animals were maintained at 22°C on a 12-h light/dark cycle with free access to water and a standard rodent diet. All animal experiments were undertaken in accordance with the National Institute of Health's “Guide for the Care and Use of Laboratory Animals,” with the approval of the Scientific Investigation Board of the Second Military Medical University.

### Cell culture

C2C12 cells (mouse muscle; myoblast; Cat. No. GNM26) were obtained from Shanghai Institutes for Biological Sciences, Chinese Academy of Sciences and cultured in DMEM high glucose supplemented with 10% fetal calf serum at 37°C and 5% CO_2_. In a dose-effect experiment, C2C12 cells were exposed to ORM at the doses of 0, 0.1, 1, or 10 μg/ml for 2 h (Sörensson et al., 2000), then cells were harvested to detect the protein expression of p-AMPK and AMPK.

### Weight-loaded forced swimming

Mice were placed individually in a cylindrical glass tank (46 cm tall, 20 cm in diameter) of water at 21–23°C. A load consisting of a steel ring weighing 8% of each body weight was attached to the proximal end of the tail. The swimming time was measured from the time the mouse began swimming to the time it could not return to the surface of the water 10 s after sinking (Lei et al., 2016). This experiment was performed double-blindedly.

### Fatigue induction in isolated muscle

Muscle fibers group into two major categories: slow twitch type I fibers with high oxidative capacity adapted to endurance exercise, fast twitch type II fibers with low oxidative capacity adapted to sprint (Vogel et al., 2015). Due to the role of ORM in enhancing muscle endurance, slow twitch soleus muscle was adopted in the present study to explore the mechanism of ORM. Muscle fatigue was induced as previously reported (Lenman et al., 1989). Briefly, mice were sacrificed and soleus muscles were quickly isolated. One end of muscle was fixed, and the other was linked to the sensor of a biotic signal collection and processing system (MedLab-U/4CS, MeiYi, Nanjing, China). The muscle contraction was electrically evoked by trains of stimuli at 10 V lasting 5 ms and delivered each second for consecutive 3 min. The ratio of tension at 1, 2, or 3 min to the initial tension (average of the first five contractions) was expressed as the fatigue index.

### Immunoblotting

Immunoblotting analyses were performed using Odyssey Infrared Fluorescent Imaging system. Samples were lysed with M-PER protein extraction reagent (Pierce, Rockford, IL, Cat. No. 78501) supplemented with protease inhibitor mixture (CalBiochem, San Diego, Cat. No. 539137). Protein concentration was estimated using Bradford reagent and bovine serum albumin as standard. Proteins were separated through SDS-PAGE and transferred onto PVDF membranes. The membranes were then incubated in primary antibodies prepared in TBST solutions (20 mM Tris-HCl, 137 mM NaCl, 0.1% Tween-20). Detection was performed using appropriate secondary antibodies conjugated with infrared dyes 680 and 800. Equal loading of the samples was confirmed by re-probing the blots for GAPDH.

### Glycogen detection

The glycogen content from mouse soleus muscle was determined using a glycogen assay kit (Biovision, USA, Cat. No. K646). In brief, 10 mg tissue was homogenized with 200 μl dH_2_O on ice and boiled for 10 min to inactivate enzymes. The boiled sample was spun at 18,000 × g for 10 min to collect the supernatant. 25 μl supernatant was added to a 96-well plate, and the volume was adjusted to 50 μl each well with hydrolysis buffer. 2 μl hydrolysis enzyme mix was added to each well and the mixture was incubated for 30 min at room temperature. Then 50 μl reaction mix was added and the mixture was incubated for another 30 min at room temperature. Absorbance at OD 570 nm was then measured. Glycogen standard curve was concurrently prepared.

### Measurements of glycogen synthase activity

Professor Yan Chen (Institute for Nutritional Sciences, Chinese Academy of Sciences) helped to detect the activity of glycogen synthase. Briefly, mouse soleus muscle extracts were prepared by homogenizing 90–100 mg powdered muscles on ice in 400 μl of homogenization buffer (50 mM Tris-HCl pH 7.8), 100 mM NaF, 10 mM EDTA, 2 mM EGTA, and protease inhibitor cocktail [Sigma-Aldrich, St. Louis, MO, Cat. No. P8340]). The homogenates were centrifuged for 5 min at 3000 rpm, and the supernatants were retained to analyze as previously described (Zhang et al., 2014). Briefly, the supernatants were measured and adjusted to a concentration of 10 mg/ml by adding homogenization buffer. Muscle extracts (3 ul) were added to reaction cocktail I (27 ul) containing 25 mM Tris-HCl (pH 7.4), 100 mM KCl, 5 mM MgCl_2_, 0.5% Glycogen (Sigma-Aldrich, St. Louis, MO, Cat. No. G8876), 10 mM glucose-6-phosphate (G-6P), and incubated with or without 5 mM 5′-urdiphosphate-glucose (UDPG, Sigma-Aldrich, St. Louis, MO, Cat. No. U4625) at 37°C for 30 min. Then the reaction was terminated at 100°C for 3 min. After centrifugation for 5 min at 10,000 rpm, supernatants (20 ul) were mixed with reaction cocktail II (10 ul) containing 6 mM HEPES (pH 7.4), 100 mM KCl, 5 mM MgCl_2_, 2 mM PEP (Sigma-Aldrich, St. Louis, MO, Cat. No. 860077), 0.4 mM NADH (Roche Applied Science, Penzberg, Germany, Cat. No. 10107735001), 4 units pyruvate kinase/ml (Sigma-Aldrich, St. Louis, Cat. No. P1506) and 2 units lactate dehydroxylase (LDH)/ml (Sigma-Aldrich, St. Louis, MO, Cat. No. L3916). Reaction product was immediately detected by spectrophotometry at 340 nm, using a microtiter plate reader (Tecan). Then GS activity was calculated as followed: U/L = ΔA/min^*^(V^*^10^6^)/(ε^*^v^*^L). ΔA: variation of the absorbance; V: volume of the reaction system (ml); ε: Molar extinction coefficient (cm.mol); v: sample amount (ml); L: the light path length of colorimetric cuvette (cm).

### Statistical analysis

**Figure 1:**
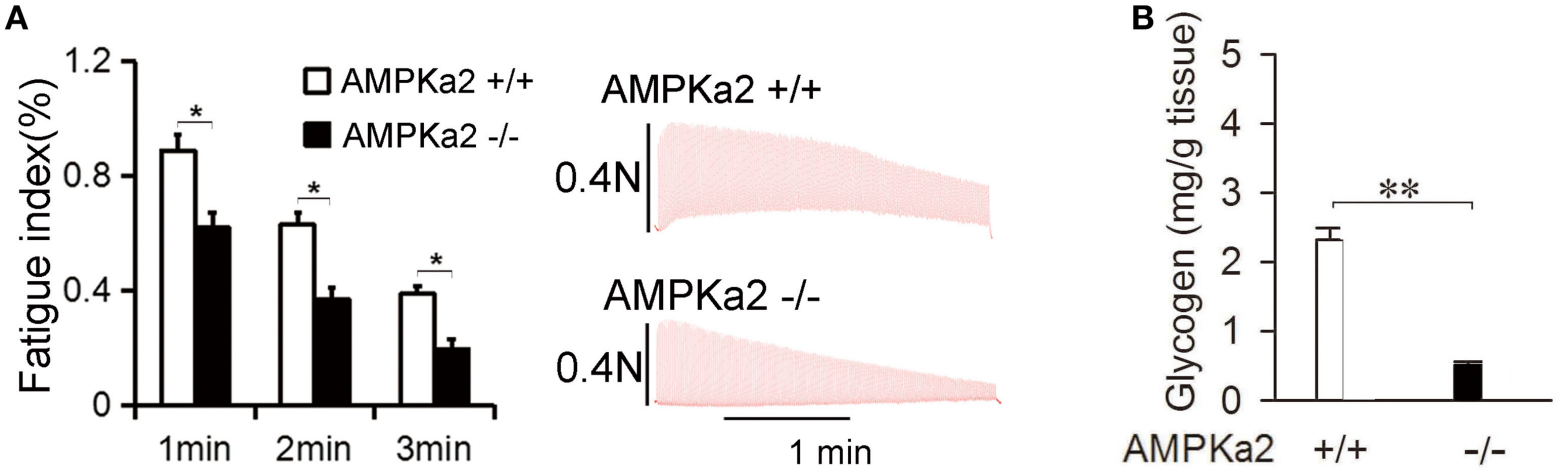
**Mice deficient in AMPKα2 have reduced muscle endurance and glycogen synthesis. (A)** Representative records of electrically evoked contractions of soleus muscle isolated from AMPKα2^+/+^ (*n* = 6) and AMPKα2^−/−^ mice (*n* = 6) for consecutive 3 min. Data are expressed as the mean ± s.d. ^*^P < 0.05 by Student's *t*-test. **(B)** Soleus muscle glycogen contents in AMPKα2^+/+^ (*n* = 6) and AMPKα2^−/−^ mice (*n* = 6). Data are expressed as the mean ± s.d. ^**^P < 0.01 by Student's *t*-test.

**Figure 2:**
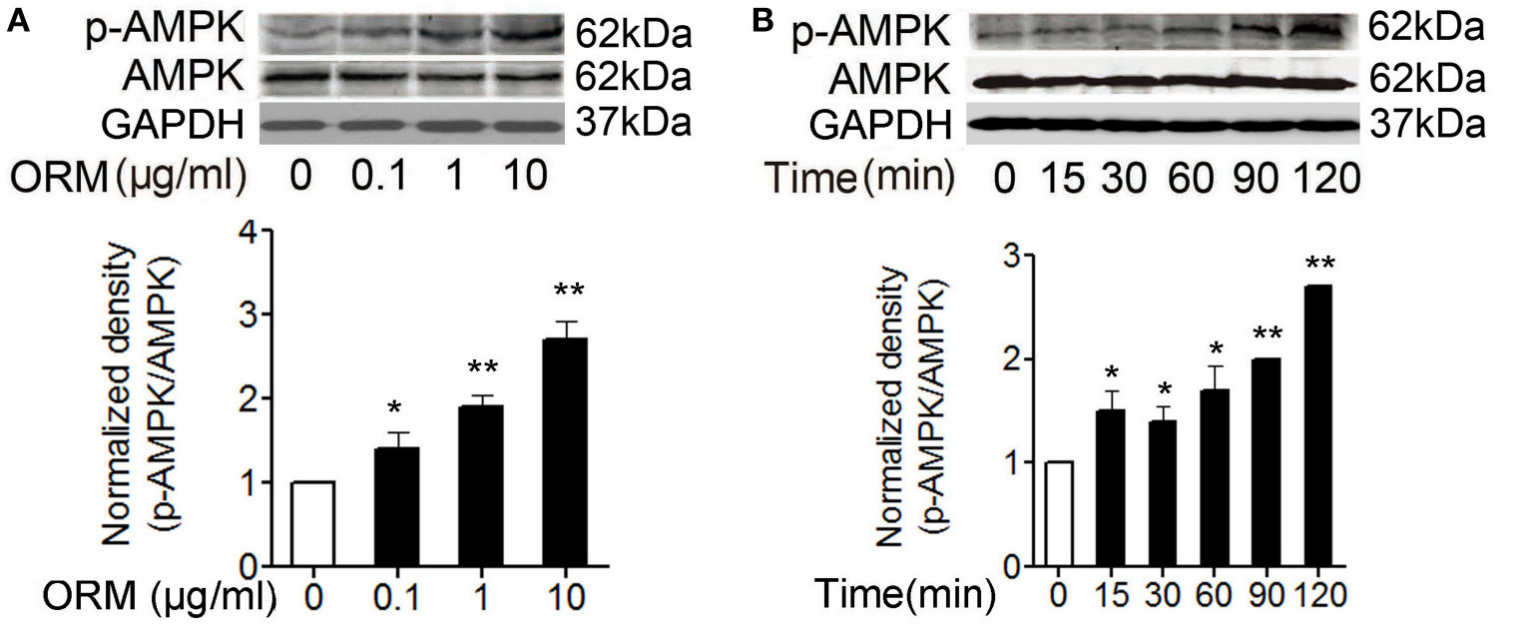
**ORM can induce the activation of AMPK in skeletal muscles. (A)** Representative western blotting of p-AMPK and total AMPK and quantification of the result in C2C12 cells treated with indicated doses of ORM for 2 h (*n* = 3 per dose). **(B)** Representative western blotting of p-AMPK and total AMPK and quantification of the result at indicated time in soleus muscle tissues of mice treated with 200 mg/kg of ORM via tail vein injection (*n* = 6 per time point). All data are expressed as the mean ± s.d.^*^P < 0.05, ^**^P < 0.01 by one-way ANOVA with Dunnett's *post-hoc* test.

**Figure 3:**
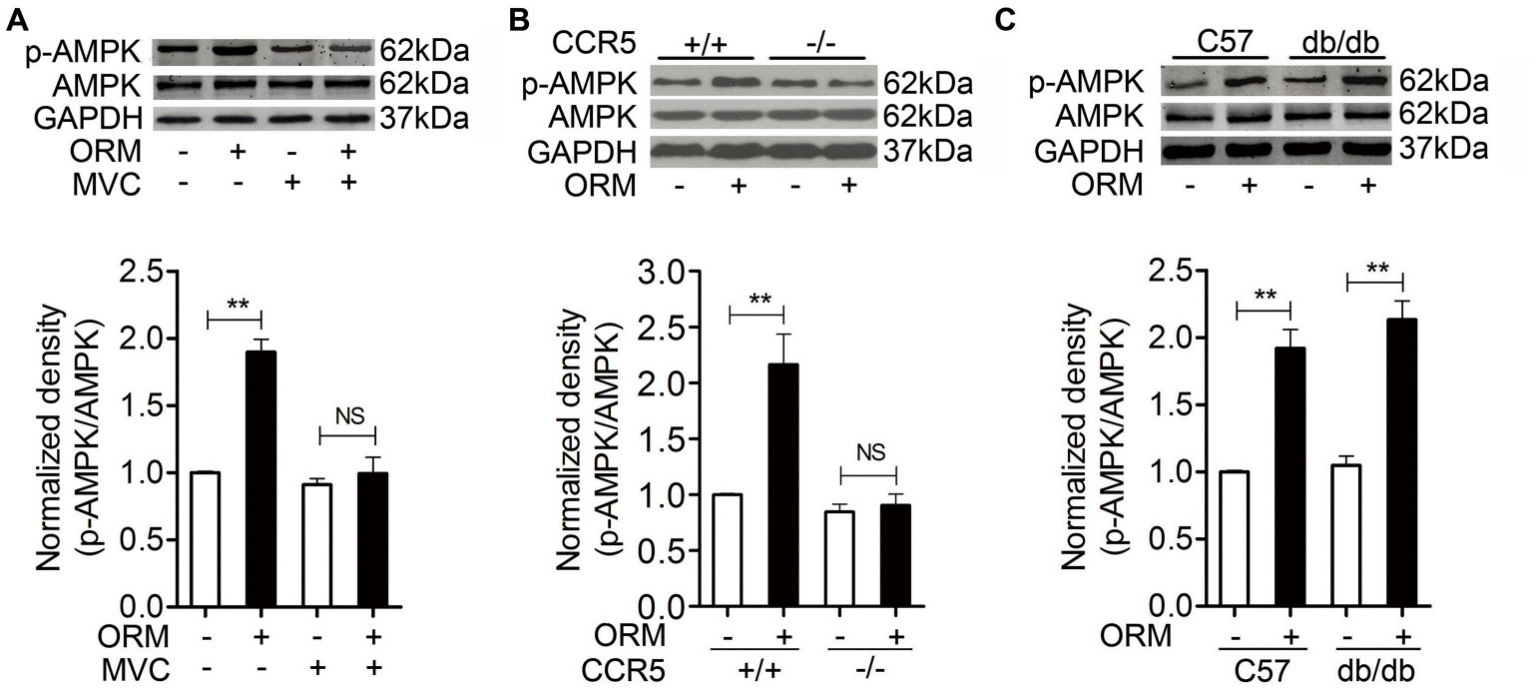
**ORM-induced AMPK activation is dependent of CCR5 in skeletal muscles. (A)** Representative western blotting of p-AMPK and total AMPK and quantification of the result in soleus muscle tissues of mice 30 min after tail-vein injection with vehicle, 200 mg/kg ORM, 200 mg/kg ORM in absence or presence of 200 mg/kg Maraviroc (MVC, gastric gavage for consecutive 3 days). *n* = 6 per group. **(B)** Representative western blotting of p-AMPK and total AMPK and quantification of the result in soleus muscle tissues of CCR5^+/+^ or CCR5^−/−^ mice 30 min after tail-injection with vehicle or 200 mg/kg ORM. *n* = 6 per group. **(C)** Representative western blotting of p-AMPK and total AMPK and quantification of the result in soleus muscle tissues of C57BL/6 or db/db mice 30 min after tail-injection with vehicle or 200ṁg/kg ORM. *n* = 6 per group. All data are expressed as the mean ± s.d. NS, non-significant, ^**^P < 0.01 by two-way ANOVA with Turkey's *post-hoc* test.

**Figure 4:**
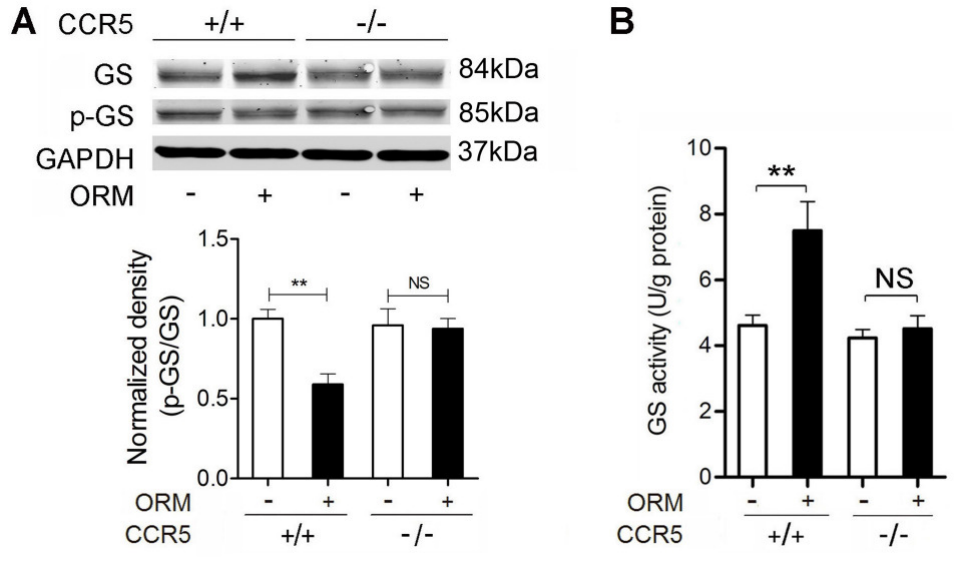
**CCR5 Mediates the Role of ORM in Promoting the Expression and Activity of Glycogen Synthase in Skeletal Muscles. (A)** Representative western blotting of soleus muscle glycogen synthase and phosphorylated glycogen synthase and quantification of the results 30 min after the treatment with vehicle or 200 mg/kg ORM (tail vein injection) in CCR5^+/+^ and CCR5^−/−^ mice. *n* = 6 per group. **(B)** The activity of glycogen synthase in soleus muscle of mice treated as mentioned in **(A)**. *n* = 6 per group. GS: glycogen synthase; p-GS: phosphorylated glycogen synthase. All data are expressed as the mean ± s.d. NS, non-significant, ^**^P < 0.01 by two-way ANOVA with Turkey's *post-hoc* test.

**Figure 5:**
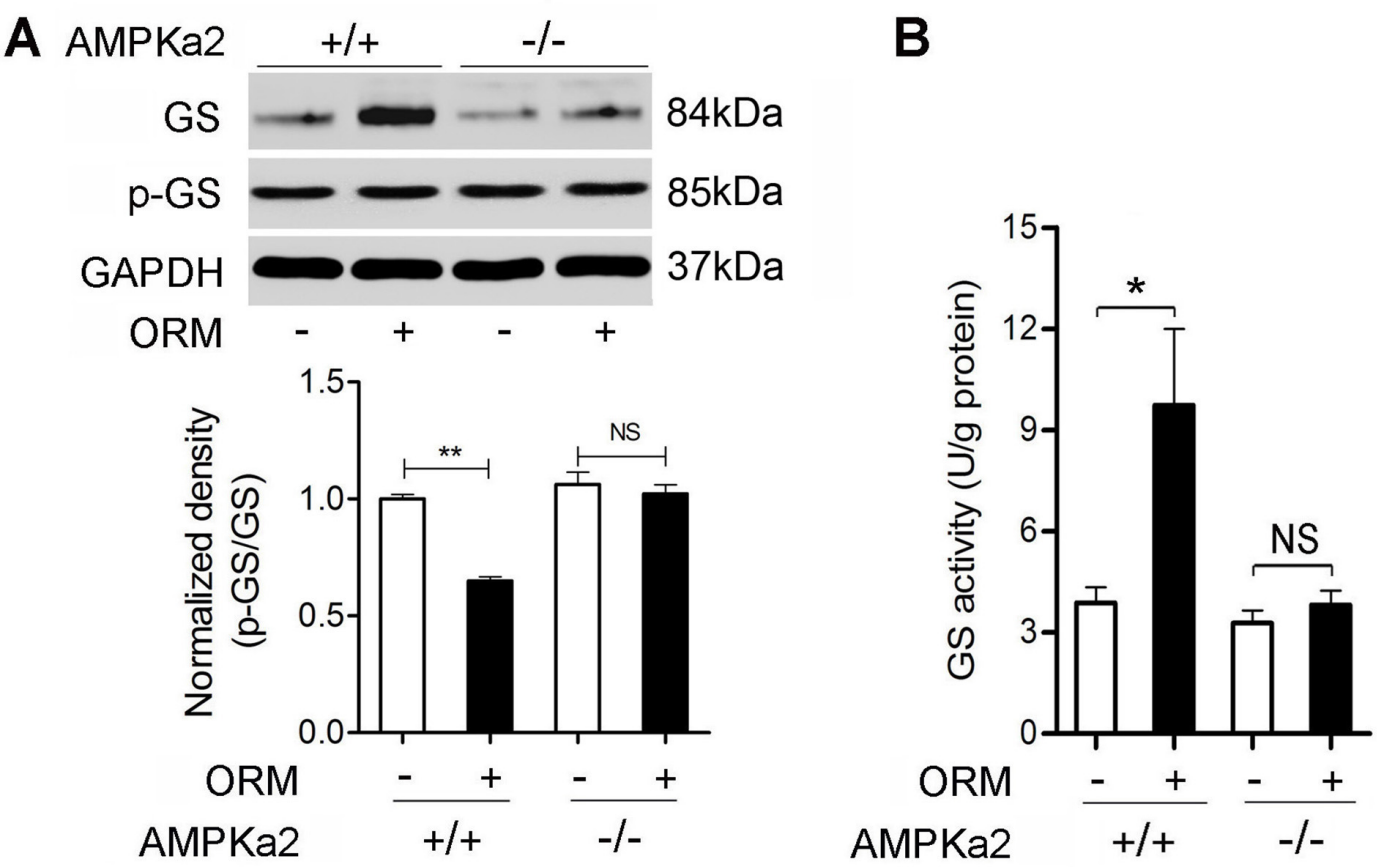
**AMPK Mediates the Role of ORM in Promoting the Expression and Activity of Glycogen Synthase in Skeletal Muscles. (A)** Representative western blotting of soleus muscle glycogen synthase and phosphorylated glycogen synthase and quantification of the results 30 min after the treatment with vehicle or 200 mg/kg ORM (tail vein injection) in AMPKα2 ^+/+^ or AMPKα2^−/−^ mice. *n* = 6 per group. **(B)** The activity of glycogen synthase in soleus muscle of mice treated as mentioned in **(A)**. *n* = 6 per group. GS: glycogen synthase; p-GS: phosphorylated glycogen synthase. All data are expressed as the mean ± s.d. NS, non-significant, ^*^P < 0.05, ^**^P < 0.01 by two-way ANOVA with Turkey's *post-hoc* test.

**Figure 6:**
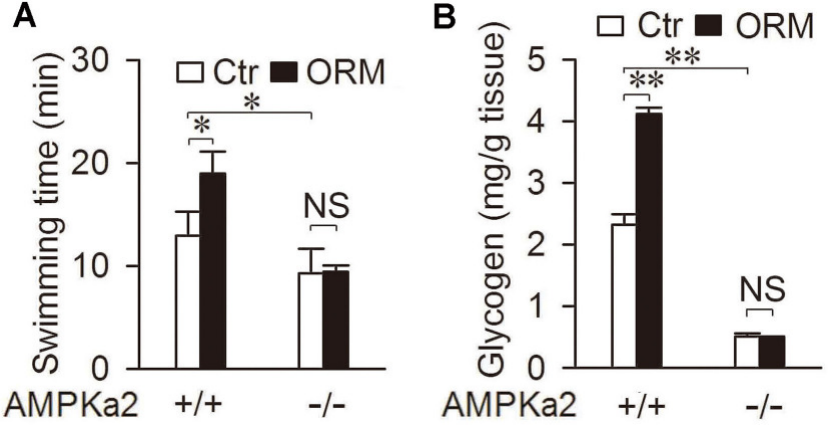
**AMPK mediates the anti-fatigue and glycogen-storage action of ORM. (A)** Swimming time of AMPKα2^+/+^ or AMPKα2^−/−^ mice 30 min after tail-vein injection with vehicle (Ctr) or 200 mg/kg ORM. *n* = 6 per group. **(B)** Muscle glycogen content 30 min after tail-vein injection with vehicle or 200 mg/kg ORM in AMPKα2^+/+^ or AMPKα2^−/−^ mice. *n* = 6 per group. All data are expressed as the mean ± s.d. NS, non-significant, ^*^P < 0.05, ^**^P < 0.01 by two-way ANOVA with Turkey's *post-hoc* test.

Data were expressed as mean ± s.d. Statistical analyses were performed using Student's *t*-test when comparing two samples with equal variance (Figure 1) or one-way ANOVA with Dunnett's *post-hoc* test for the comparison of multiple treatments to controls (Figure 2). In Figures 3–**6**, statistical evaluation was performed by two-way ANOVA. When ANOVA revealed significant differences, a *post hoc* test was used to correct for multiple comparisons (Turkey's test). Differences between groups were considered statistically significant at *P* < 0.05.

## Results

### Mice deficient in AMPKα2 have reduced muscle endurance and glycogen synthesis

We first testified whether AMPKα2 is involved in the regulation of muscle endurance and glycogen storage. Isolated mouse soleus muscle was utilized to electronically induce fatigue *in vitro*. As shown in Figure 1A, AMPKα2 knockout significantly decreased the fatigue index at 1, 2, and 3 min of the stimulation, suggesting that AMPK may directly enhance muscle endurance. Consistent with previous reports (Andreelli et al., 2003), AMPKα2 knockout mice have significantly decreased muscle glycogen content when compared to that in their wild littermates (Figure 1B). We suspected that AMPK may mediate the effect of ORM/CCR5 in enhancing muscle endurance and glycogen synthesis.

### ORM can induce the activation of AMPK in skeletal muscles

We further detected whether ORM can induce the activation of AMPK in the skeletal muscles. It has been shown that phosphorylation at T172 significantly increases AMPK activity and may also be used as a readout for its activation (Hawley et al., 1996). We found in myoblast cell C2C12, 0.1–10 μg/ml of ORM treatment for 2 h resulted in AMPK phosphorylation at T172 in a dose-dependent manner (Figure 2A). In mice injected with 200 mg/kg of ORM via tail-vein, serum ORM concentration peaked at 15 min post-injection then gradually declined (Lei et al., 2016, see Figure S5), and soleus muscle AMPK was phosphorylated at 15 min and the amount of phosphorylated AMPK peaked at 120 min post-injection (Figure 2B).

### ORM-induced AMPK activation is dependent of CCR5 in skeletal muscles

Since ORM could induce AMPK activation and the anti-fatigue effect of ORM was mediated through binding to CCR5 in the skeletal muscle (Lei et al., 2016), we want to figure out whether CCR5 is involved in the ORM-induced activation of AMPK. As shown in Figures 3A,B, tail-vein injection of 200 mg/kg of ORM for 2 h induced the phosphorylation of soleus muscle AMPK, which was inhibited by administration of CCR5 antagonist Maraviroc (200 mg/kg, via gastric gavage, for 3 days) and was largely absent in CCR5^−/−^mice, suggesting that activation of AMPK is a downstream event of CCR5 activation.

It is worth noting that, we previously found that the binding of ORM to C2C12 skeletal muscle cells is incompletely inhibited by the CCR5 antibody and antagonist, indicating ORM may bind to other cell surface proteins (Lei et al., 2016). We subsequently found ORM was able to bind directly to LepR in hypothalamus to regulate energy homeostasis. More interestingly, ORM also bound to LepR in C2C12 cells and induced LepR-mediated JAK2-STAT3 activation (Sun et al., 2016, see Figures S7, S8). We therefore further explored whether LepR is also involved in the AMPK activation triggered by ORM in skeletal muscle. As shown in Figure 3C, ORM still remains similar stimulatory effect in AMPK activation in the soleus muscle from LepR deficient db/db mice when compared to that in C57BL/6 mice, indicating ORM-triggered AMPK phosphorylation is not dependent of LepR.

### CCR5 mediates the role of ORM in promoting the expression and activity of glycogen synthase in skeletal muscles

Our previous results revealed that ORM engaged CCR5 to increase the glycogen content in skeletal muscle. Glycogen synthase (GS) is the rate-limiting enzyme of glycogen synthesis, and de-phosphorylated GS is the active form responsible for the synthesis of glycogen (Ferrer et al., 2003). We wondered whether GS was involved in this CCR5-activated glycogen accumulation. Western blot analysis revealed that 200 mg/kg of ORM significantly increased the expression of total GS and decreased the ratio of phosphorylated GS in total GS in the soleus muscles in wild-type mice, but not in CCR5^−/−^ mice (Figure 4A). Meanwhile, GS activity is markedly increased in soleus muscles from mice treated with ORM, which is inhibited in CCR5^−/−^ mice, suggesting that ORM promoted the GS activity through CCR5 (Figure 4B). Interestingly, the upregulation of glycogen synthase protein happened at 30 min post ORM-injection, indicating that it may be regulated as an immediate early gene at transcriptional level or affected by protein degradation system at post-transcriptional level, which is worthy of being deeply explored.

### AMPK mediates the role of ORM in promoting the expression and activity of glycogen synthase in skeletal muscles

We further wondered whether ORM/CCR5-activated-AMPK was also involved in the GS regulation. As shown in Figure 5, vein injection with 200 mg/kg of ORM for 30 min resulted in the significant increase in the expression of total GS (Figure 5A) and its activity (Figure 5B) in skeletal muscle in AMPKα2^+/+^ mice, but absent in AMPKα2^−/−^ mice, indicating this effect was mediated by AMPK pathway.

### AMPK mediates the anti-fatigue and glycogen-storage action of ORM

We have previously reported that administration of purified ORM to the normal mice could significantly extend their swimming time and increase their muscle glycogen storage via CCR5, in which deletion of CCR5 abolished the effect of ORM on fatigue and muscle glycogen (Lei et al., 2016). Since AMPK is the downstream event of ORM/CCR5 activation, we want to make sure that whether AMPK is involved in the regulation of swimming endurance and glycogen storage of ORM. Here we found that in AMPKα2 deficient mice, the ability of ORM to increase the swimming time was completely diminished and it also failed to increase muscle glycogen in these mice (Figures 6A,B). These results indicated that the CCR5-mediated AMPK activation is required for ORM to enhance muscle performance and glycogen storage.

## Discussion

Our previous study demonstrated that ORM could act on CCR5 to exert its anti-fatigue effect by increasing glycogen synthesis in skeletal muscle. However, the downstream pathway mediating this effect remained unclear. In this study, we presented the first evidence that ORM engaged CCR5 can activate AMPK in skeletal muscle, which led to the activation of GS and subsequent glycogen storage and endurance enhancement. Therefore, modulation of the ORM1/CCR5/AMPK system could be a novel strategy for therapeutic intervention of fatigue.

Leptin was also reported to activate AMPK via leptin receptor in soleus muscle (Minokoshi et al., 2002). We found ORM could bind to LepR in C2C12 cells and activate LepR-mediated JAK2-STAT3 pathway (Sun et al., 2016), but its effect of triggering AMPK phosphorylation in soleus muscle is independent of LepR as shown in Figure 3C. Interestingly, the binding of ORM to the hypothalamic GT1-7 cells was not competitively blocked by leptin, which is possibly due to their different binding site in LepR according to structural studies (Sun et al., 2016). We speculate this difference in the LepR binding site may be also responsible for their difference in the LepR-mediated AMPK activation.

We found here that ORM-mediated CCR5 activation can trigger AMPK phosphorylation at threonine 172 (T172). AMPK is activated through allosteric effect when intracellular levels of AMP and ADP are increased, leading to increased ATP production and switching off biosynthesis pathways (Hardie and Sakamoto, 2006). Activation of AMPK requires phosphorylation of T172 within the T loop segment of the catalytic α subunit (Hawley et al., 1996) by one or more AMPK kinases (AMPKKs). Recent studies have shown that LKB1 (Koh et al., 2008) and Ca^2+^/calmodulin kinase kinase [CaMKK, which could be activated by Ca^2+^ influx Woods et al., 2005; Shen et al., 2007] can serve as upstream kinases for AMPK. Given that muscle-specific LKB1 knockout mice (Sakamoto et al., 2005) show ablated AMPKα2 activity in skeletal muscle, LKB1 appears to be a major AMPK kinase in skeletal muscle. Whether CCR5-triggered AMPK phosphorylation was mediated by AMP/ATP (LKB1-dependent) or Ca^2+^-mediated CaMKK pathway in skeletal muscle should be further explored.

It has been reported that likely through the PLC-γ and Ca^2+^ pathway, CCR5 engagement activates AMPK to stimulate glycolysis (Chan et al, 2012; Lin et al., 2014). However, our present data suggested that CCR5-triggered AMPK activation was able to promote glycogen synthesis, not glycogenolysis, as a means to store energy in skeletal muscle. Actually the activation of AMPK stimulated muscle glucose transport (Huang and Czech, 2007). Once the glucose was taken up, it could be stored as glycogen or metabolized by glycolysis to generate ATP. Numerous studies showed that activation of AMPK was both involved in the pathway of glycolysis and glycogen synthesis (Barré et al., 2007; Viollet et al., 2009). The metabolic switch after AMPK activation was mainly dependent on the energy status of the cells. Here, we demonstrated that ORM/CCR5-triggered AMPK could induce glycogen storage in skeletal muscle, which is critical for the effect of ORM in the exercise performance.

Long-term/chronic activation of AMPK increases glycogen storage in skeletal (Holmes et al., 1999; Ojuka et al., 2000) and cardiac (Luptak et al., 2007) muscles. Glycogen synthase, catalyzing the addition of glucose residues to the non-reducing end of a nascent glycogen chain, has been considered as the key rate limiting enzyme for glycogen synthesis in all organs (Roach et al., 1998). Its activity was regulated through phosphorylation at multiple sites (Bollen et al., 1998). Phosphorylation tends to inactivate glycogen synthase and dephosphorylation results in its subsequent activation. Roger W et al recently found that AMPK activation causes an elevated glucose transport and accumulation of intracellular G6P, which leads to allosteric activation of GS resulting in a net increase in GS activity and excess glycogen storage in muscle cells (Hunter et al., 2011). Our present results showed that ORM/CCR5-triggered AMPK could increase the expression of total GS, decrease the ratio of phosphorylated GS in total GS, increase muscle GS activity, and be responsible for the effect of glycogen storage of ORM. It is worth noting that basal glycogen content was reduced in mice deficient in AMPKα2, while basal GS activity was not changed in mice deficient in AMPKα2 or CCR5, indicating an alternative pathway may be involved in the regulation of glycogen storage in embryo development. Moreover, a recent study further indicated that AMPK controls exercise endurance through increasing mitochondrial oxidative capacity and substrate utilization (Lantier et al., 2014). It is conceivable that the change of glucose transporter and GS activity are secondary to the enhancement of mitochondrial function, which should be further explored.

In summary, our present data showed that ORM promotes muscle glycogen accumulation via CCR5-AMPK-glycogen synthase pathway, representing a new energy homeostasis regulatory mechanism when body meets stress, and also a possible target acting as a potential antidiabetic drug due to its glycogen storage effect. Considering that ORM can increase glucose uptake in 3T3-L1 adipocytes (Lee et al., 2010) and regulate whole body energy homeostasis (Sun et al., 2016), it is conceivable that when systemic administration of ORM, these effects may also influence the direct role of ORM in muscle endurance, and the potential cross talk between them should also be explored in the future.

## Author contributions

XL and HL designed and supervised the experiments. ZQ, JW, YS, and PW carried out the experiments and data analyses. XL and HL drafted the manuscript. DS participated in the discussion and formation of the manuscript. All authors have read and approved the final manuscript.

## Funding

This work was supported by Grants from the National Natural Science Foundation of China (No. 81273606, 81473259 to XL, 81603116 to YS), and National Science and Technology Major Project (2014ZX09J14103-08C to XL).

### Conflict of interest statement

The authors declare that the research was conducted in the absence of any commercial or financial relationships that could be construed as a potential conflict of interest.
